# Impact of follow-up interval on patients with hepatocellular carcinoma after curative ablation

**DOI:** 10.1186/s12885-018-5069-z

**Published:** 2018-11-29

**Authors:** Wenwu Liu, Yun Zheng, Ruhai Zou, Jingxian Shen, Wei He, Zhiwen Yang, Yuanping Zhang, Binkui Li, Yunfei Yuan

**Affiliations:** 1 State Key Laboratory of Oncology in South China, Collaborative Innovation Center for Cancer Medicine, Sun Yat-Sen University Cancer Center, Guangzhou, China; 2 Department of Hepatobiliary Oncology, Sun Yat-Sen University Cancer Center, 651 Dongfeng Road East, Guangzhou, 510060 China; 3 Department of Ultrasound, Sun Yat-Sen University Cancer Center, Guangzhou, China; 4 Department of Medical Imaging, Sun Yat-Sen University Cancer Center, Guangzhou, China

**Keywords:** Liver cancer, Milan criteria, Overall survival, Recurrence-free survival, Surveillance, Thermal ablation

## Abstract

**Background:**

The optimal follow-up strategy after curative thermal ablation of hepatocellular carcinoma (HCC) remains unclear.

**Methods:**

We retrospectively analyzed a prospective series of 616 patients who underwent curative thermal ablation for HCC within the Milan criteria. Multivariate Cox model was used to identify independent predictive factors for recurrence; accordingly, patients were stratified into 2 groups with different relapse risks: a low-risk group (solitary tumor ≤3 cm) and a high-risk group (multiple tumors ≤3 cm or solitary tumor between 3 and 5 cm). Then, patients were classified into short- (< 4 months) or long-interval (4–6 months) surveillance groups according to follow-up intensity within the first 2 years after ablation. The overall survival (OS) of patients were compared between short- and long-interval groups in low- or high-risk groups, as well as the stage of recurrent tumors and the proportion of patients who received curative-intent retreatments.

**Results:**

In the low-risk group, 54 (83.0%) and 18 (72.0%) of patients exhibited early relapse at the Barcelona Clinic Liver Cancer (BCLC) 0/A stage in the short- and long-interval groups, respectively (*P* = 0.172); accordingly, 44 (77.2%) and 18 (81.8%) of patients received curative-intent retreatment (*P* = 0.086) after recurrence. Hence, 5-year OS was similar between short- and long-interval groups (80.4% vs. 77.5%, *P =* 0.400) in low-risk patients. However, in the high-risk group, patients with a short interval exhibited early relapse more frequently at the BCLC 0/A stage (83% vs. 72%, *P* = 0.028), with a trend showing that the corresponding proportion of patients who received curative-intent retreatment greater than that in the long-interval group (64.2% vs. 37.5%, *P* = 0.087). Moreover, the short-interval group showed better 5-year OS than the long-interval group in high-risk patients (69.9% vs. 42.7%, *P* = 0.020).

**Conclusions:**

Compared to a short surveillance interval, a long surveillance interval does not reduce OS in low-risk patients; however, a long surveillance interval compromises OS in high-risk patients.

## Background

Hepatocellular carcinoma (HCC) is one of the leading causes of cancer deaths worldwide, especially in less developed countries, with China alone accounting for approximately 50% of the total number of cases and deaths [1]. As one of the curative-intent treatments and the standard of care for patients with small HCC, radiofrequency ablation (RFA) has been widely used in the past decade [2]. Yu et al. found that both microwave ablation (MWA) and RFA were suitable first-line options for early stage HCC in a large-sample randomized controlled trials (RCT) [3]. However, there are more than half of patients will face tumor relapse in the first 2 years after ablation, with a 37.4% 3-year recurrence-free survival [4]. Although tumor recurrence is common and there is a consensus that earlier identification of recurrence may facilitate patient eligibility for retreatment, especially for curative-intent retreatment, current guidelines seldom address the optimal surveillance interval for post-ablation of HCC and only with generic recommendations [5–8]. As the largest cancer center in southern China, our hospital established a prospective electronic database tracking all treated cancer patients, and these data provide a unique opportunity to explore an optimal follow-up strategy for post-ablation of HCC.

## Methods

### Patient cohort

An electronic medical record system, including a prospective follow-up database, has been maintained at Sun Yat-sen University Cancer Center since 2002 to track all cancer patients treated in the center, including HCC patients. In this prospective cohort study, all HCC patients who were initially treated with curative-intent thermal ablation (RFA/MWA) between January 2002 and January 2017 were identified. Ablation procedures were performed as described in our previous study [9]. A total of 856 patients were included using the following inclusion criteria: (a) Tumors within the Milan criteria; (b) Tumors exceeding the Milan criteria that were downgraded to within the Milan criteria by pre-ablation transcatheter arterial chemoembolization (pre-TACE); (c) No radiological evidence of major portal/hepatic vein branch invasion; (d) No extrahepatic metastasis; and (e) Child-Pugh A or B disease. Two hundred and forty patients were excluded based on the following exclusion criteria: (a) Patients without image (computed tomography (CT)/magnetic resonance imaging (MRI)) assessment of technique efficacy within 3 months after ablation; (b) Patients without complete ablation (complete ablation was defined as no enhancement in the ablated area on the first CT/MRI scan); and (c) Complete ablation achieved by multiple ablation courses. Finally, 616 patients were included in this study. The study protocol conformed to the ethical guidelines of the 1975 Declaration of Helsinki, and the Ethics Committee of Sun Yat-sen University Cancer Center approved this study and written informed consent was obtained before treatment.

### Surveillance data collection

The first follow-up visit, approximately 1 month after ablation (3 months at most), was not regarded as the start of the surveillance regimen because it was performed to assess ablation efficacy. Follow-up visits were performed every 3–6 months until death or dropout from the surveillance program and each follow-up consisted of a physical examination, serum AFP test and at least one imaging examination (CT/MRI). To study the influence of surveillance interval on overall survival (OS), we took advantage of the known variability in patient compliance with the above follow-up strategy.

The patients were stratified into short- (< 4 months) or long-interval (4–6 months) groups by comparing the number of their actual follow-ups with the number of their expected follow-ups by the time of the last follow-up or when recurrence was detected [10]. As shown in the following example, one patient who had received 4 follow-ups within the 12 months after the first clinic visit was classified into the short-interval group because the actual number of follow-ups (4) was greater than the minimal expected number of follow-ups for the short-interval group (3). In contrast, one patient who had received 2 follow-ups within the 12 months after the first clinic visit was classified into the long-interval group because the actual number of follow-ups (2) was less than the maximal expected number of follow-ups for the long-interval group (3).

As all cirrhotic patients require ultrasound examination every 6 months [11, 12], it makes sense to recommend enhanced surveillance in patients after ablation for HCC. Hence, patients with surveillance intervals longer than 6 months (*n* = 171) were used only in the exploration of relapse risk factors, not in the surveillance analysis, as such scenarios were deemed irregular.

### Recurrence data collection

All consecutive post-ablation examinations performed for follow-up were retrieved from the electronic database. The recurrence data were used to explore the relapse pattern after ablation for HCC [13, 14]. From the RFS curve and the probability density plot, we found that more than half of patients (53.1%) experienced recurrence within 2 years after ablation, which is known as early recurrence. From the hazard rate curve, we found that the hazard of relapse reached its peak in the first year, decreased to its nadir in the third year and then increased again, which is considered late recurrence [15]. Late recurrences of HCC are thought to stem from de novo lesions related to the underlying chronic liver disease, persisting for the duration of the patient’s lifespan [11]. Thus, it makes sense to focus on surveillance during the first 2 years after ablation to detect early recurrence at a potentially more treatable stage. The characteristics of recurrent tumors and subsequent retreatments were also recorded. Curative-intent retreatments included resection, repeat ablation; other retreatments, such as TACE and biotherapy, were deemed as palliative [16].

### Relapse risk classification of HCC patients after ablation

Variables with *P* value less than 0.10 in univariate analysis were introduced into the multivariate Cox model to identify independent prognostic factors for recurrence, and finally tumor number and tumor size were identified. Accordingly, patients with a single tumor of 3 cm or smaller were classified into the low-risk group, the remainder were classified into the high-risk group. Then, recurrence-free survival (RFS) were compared between the low- and high-risk groups.

### Statistical analysis

Continuous variables were compared using the independent sample t-test and the Mann-Whitney U-test where appropriate. Binary and ordinal categorical variables were compared using the chi-squared test (Fisher’s exact test if necessary) and the Kruskal-Wallis test, respectively. OS curves were constructed and compared using the Kaplan-Meier method and log-rank test, respectively. All statistical analyses were performed using the R statistical package (R software version 3.3.2; R Foundation for Statistical Computing, Vienna, Austria) [17]. *P* values less than 0.05 were considered statistically significant, and all tests were two-tailed, except the tests for the BCLC stage of recurrent tumors, which were one-tailed because the stage of recurrent tumors in the short-interval group was not greater than that in the long-interval group.

## Results

### Stratification of patients by recurrence risk

**Fig. 1:**
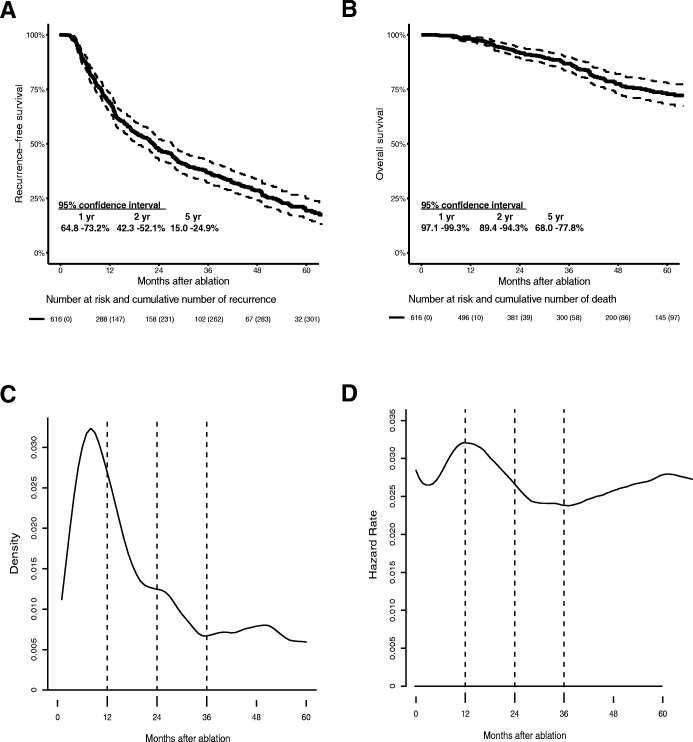
The OS and RFS curves with 95% CIs and risk tables and the recurrence patterns for all HCC patients treated with ablation. **a** The RFS of 616 patients. The 1, 2- and 5-year RFS rates were 68.9, 46.9 and 19.3%, respectively. **b** The OS of 616 patients. The1-, 2- and 5-year OS rates were 98.2, 91.8 and 72.7%, respectively. **c** The probability density plot of recurrence showed that relapse cases centered in the first 2 years after ablation. **d** The hazard rate of recurrence curve showed that the recurrence hazard peaked during the first 2 years after ablation

We identified 616 patients who were initially treated with RFA/WMA of curative intent and the characteristics are shown in Table 1. The median follow-up period was 34.4 months (range 2–171 months). Overall, 319 (51.8%) patients developed recurrence, with 2-year RFS rate of 46.9%, and 5-year RFS rate of 19.3% (Fig. 1a), and 116 (11.8%) patients died, with 2-year OS rate of 91.8%, and 5-year OS rate of 72.7% (Fig. 1b). Relapse cases centered in the first 2 years after ablation (Fig. 1c), and recurrence hazard peaked in the first 2 years after treatment (Fig. 1d).

**Table 1:** Baseline characteristics of all HCC patients

Characteristics	Value
Patients	616
Pre-TACE	145 (23.5)
Age (yrs.)	56.0 (18.0)
Male	556 (90.3)
Ablation	
Radiofrequency	469 (76.1)
Microwave	147 (23.9)
Tumor number	
Single	499 (81.0)
Multiple	117 (19.0)
Tumor size (cm)	
≤ 3	473 (76.8)
> 3	143 (23.2)
Risk area	159 (25.8)
AFP ≥200 ng/ml	187 (30.4)
Hb (10^9^/L)	142.0 (22.3)
PLT (10^9^/L)	121.0 (90.0)
RBC (10^9^/L)	4.6 (0.8)
WBC (10^9^/L)	5.2 (2.5)
ALB (g/L)	41.5 (6.0)
ALT (U/L)	35.2 (26.8)
AST (U/L)	33.6 22.57)
TBIL (μmol/L)	15.0 (20.4)
Child-Pugh grade	
A	594 (96.4)
B	22 (3.6)
PT (seconds)	12.3 (1.8)
Viral hepatitis	574 (93.2)
Cirrhosis	503 (81.7)

*Abbreviations*: *TACE* Transcatheter arterial chemoembolization, *AFP* alpha-fetoprotein, *Hb* Hemoglobin, *PLT* platelet, *RBC* red blood cell, *WBC* white blood cell, *ALB* albumin, *ALT* alanine aminotransferase, *AST* aspartate aminotransferase, *TBIL* total bilirubin, *PT* prothrombin time

Values are presented as the median (IQR) or n (%)

**Fig. 2:**
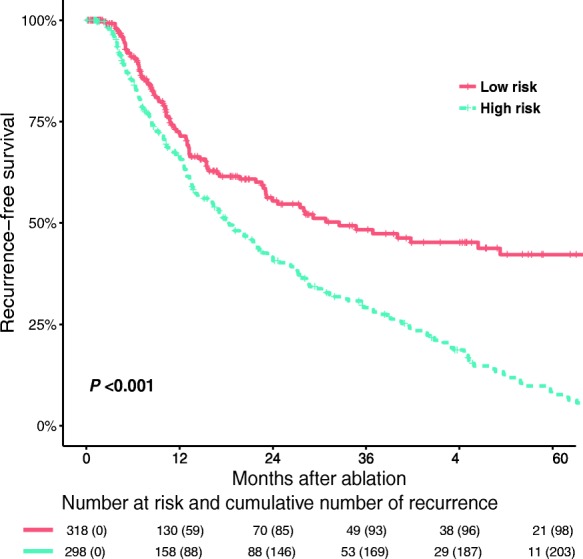
The recurrence risk classification of entire patients. The RFS curve of high- and low-risk patients (HR = 1.98; 95% CI, 1.56 to 2.51; *P* < 0.001)

At multivariate analysis, tumor number (HR = 1.32; 95% CI, 1.00–1.73, *P* = 0.048) and tumor size (HR = 1.26; 95%CI, 1.11–1.43, *P* < 0.001; Table 2) were identified as risk factors for relapse, and accordingly, patients were classified into 2 groups with different relapse risks: the low-risk group (single tumor ≤3 cm; *n* = 318 patients) and the high-risk group (single tumor between 3 and 5 cm or 2–3 tumors ≤3 cm; *n* = 298 patients). The RFS curve of the high- and low-risk groups (HR = 1.98; 95% CI, 1.56 to 2.51, *P* < 0.001) was distinguished in Fig. 2.

**Table 2:** Recurrence-free survival prognostic factors

Variable	Recurrence-free survival			
	Univariate		Multivariate	
	HR (95% CI)	*P*	HR (95% CI)	*P*
Age	1.24 (0.98, 1.56)	0.068	1.19 (0.94, 1.51)	0.142
Male	0.94 (0.64, 1.36)	0.732		
Tumor number	1.47 (1.13, 1.91)	< 0.001	1.32 (1.00, 1.73)	0.048
Tumor size	1.32 (1.18, 1.49)	< 0.001	1.26 (1.11, 1.43)	< 0.001
Risk area	1.20 (0.94, 1.53)	0.138		
AFP ≥200 ng/mL	1.25 (0.99, 1.57)	0.067	1.25 (0.98, 1.58)	0.067
Pre-TACE	1.23 (0.96, 1.58)	0.097	1.07 (0.82, 1.40)	0.606
PLT < 100 × 10^9^/L	1.28 (1.02, 1.60)	0.032	1.18 (0.93, 1.84)	0.169
RBC < 4.3 × 10^9^/L	1.00 (0.79, 1.26)	0.975		
WBC < 4.0 × 10^9^/L	1.20 (0.93, 1.56)	0.161		
ALB < 35 g/L	1.48 (1.07, 2.06)	0.019	1.30 (0.92, 1.84)	0.133
ALT < 50 U/L	1.00 (0.78, 1.28)	0.990		
AST < 40 U/L	1.07 (0.85, 1.35)	0.578		
TBIL > 17.1 μmol/L	1.01 (0.96, 1.07)	0.695		
PT > 16.5 s	0.80 (0.40, 1.63)	0.545		
Cirrhosis	1.12 (0.88, 1.57)	0.263		

*Abbreviations*: *AFP* alpha-fetoprotein, *TACE* transcatheter arterial chemoembolization, *PLT* platelet, *RBC* red blood cell, *WBC* white blood cell, *ALB* albumin, *ALT* alanine aminotransferase, *AST* aspartate aminotransferase, *TBIL* total bilirubin, *PT* prothrombin time

Variables with *P* value < 0.10 at univariate analysis were retained for multivariate analysis

### Surveillance patterns

Excluding the 171 patients with irregular surveillance, 445 patients with regular follow-up were classified into 2 groups: the short-interval group (interval < 4 months; *n* = 344 patients) and the long-interval group (interval between 4 and 6 months; *n* = 101 patients), and the mean surveillance interval was 2.58 months and 4.86 months, respectively. Finally, 199 (75.7%) and 64 (24.3%) patients underwent short- and long-interval surveillance in the low-risk group, and 145 (79.7%) and 37 (20.3%) patients underwent short- and long-interval surveillance in the high-risk group. The distribution of surveillance interval in the low- and high-risk groups was similar (*P* = 0.381), and the pertinent baseline clinic characteristics are shown in Table 3.

**Table 3:** Baseline characteristics of HCC patients with regular surveillance

Characteristics	Low risk group			High risk group		
	Short interval	Long interval	*P*	Short interval	Long interval	*P*
Patients	199 (75.7)	64 (24.3)		145 (79.7)	37 (20.3)	
Pre-TACE	22 (11.1)	11 (17.2)	0.284	71 (49.0)	12 (32.4)	0.106
Age (yrs.)	55.0 (17.5)	54.0 (19.5)	0.962	56.0 (14)	61.0 (21)	0.058
Male	177 (88.9)	61 (95.3)	0.206	135 (93.1)	35 (94.6)	1.000
Tumor number			1.000			0.971
Singular	199 (100.0)	64 (100.0)		69 (47.6)	18 (48.6)	
Multiple	–	–		76 (52.4)	19 (51.4)	
Tumor size						0.631
≤ 3 cm	199 (100.0)	64 (100.0)		34 (37.2)	16 (43.2)	
> 3 cm	–	–		91 (62.8)	21 (56.8)	
Risk area	50 (25.1)	14 (21.9)	0.719	39 (26.9)	9 (24.3)	0.914
AFP ≥200 ng/ml	67 (33.7)	10 (15.6)	0.009	86 (59.3)	20 (54.1)	0.695
Hb (10^9^/L)	143.0 (2.80)	145.5 (19.2)	0.331	142.0 (21.2)	142.0 (22.1)	0.367
PLT (10^9^/L)	131.0 (96.0)	129.5 (95.9)	0.896	105.1 (85.0)	132.0 (98.0)	0.452
RBC (10^9^/L)	4.5 (0.8)	4.8 (0.8)	0.013	4.6 (0.8)	4.5 (0.9)	0.556
WBC (10^9^/L)	5.3 (2.5)	5.4 (2.5)	0.579	5.2 (2.5)	5.80 (0.9)	0.357
ALB (g/L)	41.9 (7.9)	42.5 (5.6)	0.671	41.2 (5.8)	40.3 (6.7)	0.804
ALT (U/L)	34.1 (23.4)	37.2 (29.5)	0.229	37.1 (29.1)	36.0 (25.3)	0.773
AST (U/L)	32.3 (20.0)	33.5 (20.0)	0.802	35.7 (22.3)	35.5 (28.6)	0.868
TBIL (μmol/L)	14.4 (9.0)	15.2 (6.8)	0.678	16.0 (11.8)	14.3 (5.2)	0.137
Child-Pugh grade			0.732			0.348
A	191 (96.0)	44 (95.3)		138 (95.2)	44 (100.0)	
B	8 (4.0)	3 (4.7)		7 (4.80)	0 (0.00)	
PT (seconds)	12.3 (1.7)	12.1 (1.6)	0.135	12.3 (1.8)	12.4 (1.8)	0.837
Viral hepatitis	184 (92.5)	59 (92.2)	1.000	137 (94.5)	34 (91.9)	0.838
Cirrhosis	159 (79.9)	51 (79.7)	1.000	125 (86.2)	26 (70.3)	0.040

*Abbreviations*: *TACE* Transcatheter arterial chemoembolization, *AFP* alpha-fetoprotein, *Hb* Hemoglobin, *PLT* platelet, *RBC* red blood cell, *WBC* white blood cell, *ALB* albumin, *ALT* alanine aminotransferase, *AST* aspartate aminotransferase, *TBIL* total bilirubin, *PT* prothrombin time

Values are presented as the median (IQR) or n (%)

### Comparison of early recurrence and retreatment

The 190 patients who underwent regular follow-up with early relapse were stratified into low- and high-risk groups. In the low-risk group, 54 (83%) patients with a short surveillance interval and 18 (72%) patients with a long surveillance interval were found to be relapsed with tumors at the BCLC 0/A stage (*P* = 0.172), and 44 (77.2%) patients and 18 (81.8%) patients received curative-intent retreatment, retrospectively (*P* = 0.886) (Table 4). However, in the high-risk group, patients in the short-interval group were found to have relapse more frequently at the BCLC 0/A stage than patients in the long-interval group (83% vs. 72%, *P* = 0.028), consequently, the proportion of patients who received curative-intent retreatment was higher for short-interval group (64.2% vs. 37.5%, *P =* 0.087), though with no significant difference (Table 4).

**Table 4:** Clinical characteristics of early recurrence

Characteristics	Low risk group			High risk group		
	Short interval	Long interval	*P*	Short interval	Long interval	*P*
Patients	65	25		79	21	
Relapse location (%)			0.868			0.383
Local	26 (40.6)	11 (44.0)		29 (37.2)	5 (23.8)	
Intrahepatic distant	37 (57.8)	14 (56.0)		47 (60.3)	15 (71.4)	
Extrahepatic	1.0 (1.6)	0.0 (0.0)		2 (2.6)	1 (4.8)	
Intrahepatic tumor number	1.0 (1.0)	1.5 (1.0)	0.811	1.0 (1.0)	2.0 (1.0)	0.229
Intrahepatic tumor size (cm)	1.7 (1.5)	1.9 (1.9)	0.718	1.9 (1.5)	1.6 (1.3)	0.797
Curative intent (%)	44.0 (77.2)	18.0 (81.8)	0.886	43 (64.2)	6 (37.5)	0.087
BCLC (%)			0.172			0.028
0	19 (29.2)	6 (24.0)		15 (19.0)	2 (9.5)	
A	35 (53.8)	12 (48.0)		46 (58.2)	10 (47.6)	
B	7 (10.8)	5 (20.0)		8 (10.1)	3 (14.3)	
C & D	4 (6.2)	2 (8.0)		10 (12.7)	6 (28.6)	

*Abbreviations*: *BCLC* Barcelona clinic liver cancer stage

Values are presented as the median (IQR), or n (%)

### Comparison of survival

**Fig. 3:**
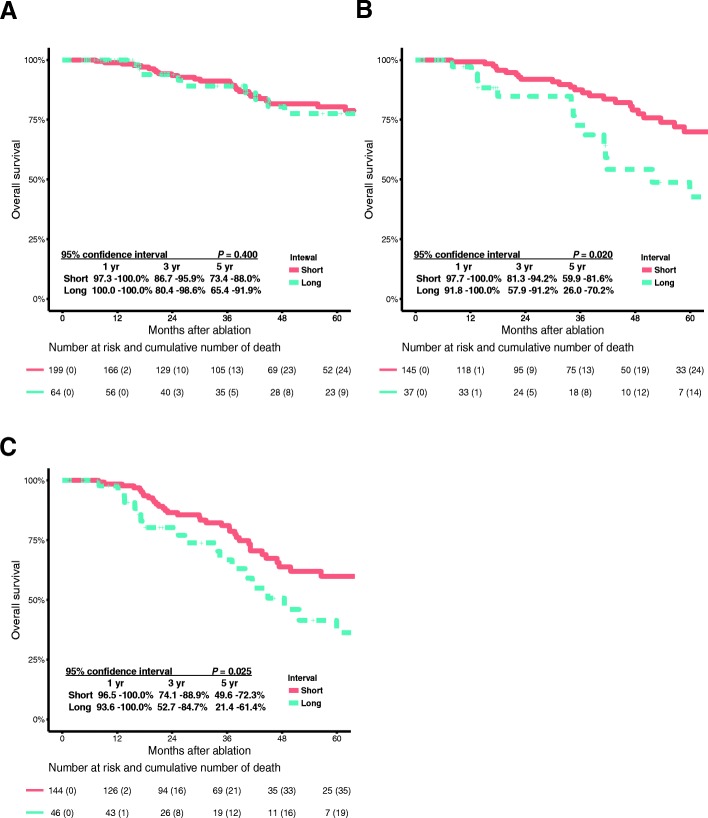
Overall survival curves with risk tables of patients in low- or high-risk group with short or long surveillance interval. **a** The OS of 263 low-risk patients with short or long surveillance. **b** The OS of 182 high-risk patients with short or long surveillance. **c** The OS of the 190 patients with early relapse with short or long interval

The 1-, 3-, 5-year OS of low-risk patients with short or long interval were 98.9, 91.2 80.4 and 100.0%, 89.1, 77.5%, respectively (*P* = 0.400) (Fig. 3a). The 1-, 3-, 5-year OS of high-risk patients with short or long interval were 99.2, 87.5, 69.9 and 97.1%, 72.7, 42.7%, respectively (*P* = 0.020) (Fig. 3b). Considering the 190 patients with early relapse, the 1-, 3-, 5-year OS of low-risk patients with short or long interval were 98.5, 81.1, 59.9 and 97.8%, 66.8, 36.3%, respectively (*P* = 0.025) (Fig. 3c).

## Discussion

There is a lack of consensus as to follow-up regimen after treatment of HCC, especially for post-ablation. Hence, we prudently defined the follow-up interval according to the following three guidelines. The ESMO proposes an intensive surveillance regimen after resection or RFA: CT/MRI scans of liver every 3 months [5]; and the Italian Association for the Study of the Liver recommends a moderate surveillance regimen for resection or ablation: AFP measurements and US examination of the liver every 3–4 months and CT/MRI scans of the liver every 6 months [7]. Additionally, patients with a follow-up interval of less than 2 months were excluded, as such scenarios were deemed irregular and likely to influence the outcome. Therefore, the short interval of follow-up was set as 2–4 months.

However, the NCCN proposes a less intensive surveillance strategy in the first 2 years after resection (no recommendation for ablation): MRI/CT scans for liver assessment every 3–6 months combined with AFP testing [6]. As all cirrhotic patients require ultrasound examination every 6 months to screen for HCC [11, 12], it makes sense to recommend enhanced surveillance in patients after ablation for HCC. Therefore, the upper limit of the follow-up interval was set to not greater than 6 months and the long interval of follow-up was defined as 4–6 months.

Since ablation is associated with higher rate of tumor recurrence than surgery [18], and in fact, Hyder et al. analyzed the surveillance patterns following HCC treatment using the Surveillance, Epidemiology and End Results linked Medicare database and found that the ablation was associated with higher intensity of follow-up imaging than resection (OR = 2.77, *P* = 0.01) [19], it might be more appropriate to establish an specific follow-up regimen for its own. Hence, our study supported an enhanced surveillance strategy that can discover recurrent tumors at an early and potentially more treatable stage for high-risk patients, with OS improvement, and we also suggested a less intensive and cost-effective follow-up strategy for low-risk patients without compromising OS.

After patients with regular follow-up were stratified into short- or long-interval groups, we found that the OS in the short-interval group seems better than that in the long-interval group, although without significance. Then, we classified patients into 2 groups with different risk for recurrence based on the hypothesis that patients with different relapse risk require different scanning schedules [20]. Tumor number and tumor size were identified as significant risk factors for post-ablation relapse, which were consistent with those reported in the literature [21–23]. Patients with a single tumor ≤3 cm were classified into the low-risk group, while patients with a single tumor between 3 and 5 cm or 2–3 tumors≤3 cm were classified into the high-risk group. Patients were then further classified according to both relapse risk and surveillance interval to determine the optimal surveillance interval.

In the low-risk group, the OS was similar between the short- and long-interval group. In addition, tumor size, tumor number and the BCLC stage of recurrent tumors were also similar between the short- and long-interval groups as well as the corresponding proportion of patients who received curative-intent retreatment, which was consistent with the findings of Liu et al. [20]. The data above support the notion that prolonging the surveillance interval to 4–6 months in the low-risk group does not reduce the efficacy of detecting HCC recurrence or compromise OS.

In the high-risk group, although the difference of recurrent tumor size and recurrent tumor number was not significant, the BCLC stage of recurrent tumors was earlier in the short-interval group than in the long-interval group. There was also a trend suggesting that the corresponding proportion of patients who received curative-intent retreatment was greater than that in the long-interval group. Moreover, the OS of the short-interval group was better than that of the long-interval group for high-risk patients, which was likely because intensive surveillance can identify recurrent disease at an earlier time point and at a more treatable stage. Similarly, Cucchetti et al. found that 5-year OS increased from 52.6 to 65.8% for HCC patients after resection, which the authors attributed to more curative-intent retreatments (increased from 22.2 to 36.9% of patients in the intensive surveillance group) after they initiated a more intensive surveillance program [24]. In conclusion, patients with a short follow-up interval in the first 2 years showed better OS in the high-risk group than those with a long follow-up interval.

Additionally, for low-risk HCC patients, the proposed risk-based surveillance strategy reduces the total imaging scans, with the accompanied reduction in blood tests, radiation dose, and attendant traffic and accommodation costs. Analogous to patients with HCC after liver transplantation (LT), Liu et al. [25] found that in the first 5 years after liver transplantation, RFS was not significantly different when the imaging interval was extended from the current every 3 months to every 6 months. In addition, Ladabaum U et al. [26] built a Markov model and concluded that screening for 2 years in only those whose explant pathology exceeding the Milan criteria (high relapse risk) may be relatively cost-effective, further supporting the notion that patients with different risk rates require different scanning schedules.

The conclusion of this study could be influenced by the potential confounding bias since the present study was a non-randomized controlled trial (RCT), and future RCTs are warranted to validate our study. Additionally, our study was also limited by a lack of external validation for the relapse risk stratification model. Although tumor size combined with tumor number can stratify patients with HCC into low or high groups with different relapse risk, it might be more effective to discriminate high-risk patients from low-risk patients using additional factors related to tumor biology, such as CpG methylation signatures [27] and microRNAs [28], and it also makes sense to take the risk factors during the follow-up period into consideration such as antiviral therapy.

## Conclusion

In summary, it is recommended to perform at least 3 follow-ups every year for high-recurrence-risk patients in the first 2 years after ablation, while in low-recurrence-risk patients, it is recommended to perform 2–3 follow-ups every year.

## Abbreviations

AFP: Alpha fetoprotein
BCLC: Barcelona Clinic Liver Cancer
CI: Confidence interval
CT: Computed tomography
ESMO: European Society for Medical Oncology
HCC: Hepatocellular carcinoma
HR: Hazard ratio
MRI: Magnetic resonance imaging
MWA: Microwave ablation
NCCN: National Comprehensive Cancer Network
OS: Overall survival
RCT: Randomized controlled trials
RFA: Radiofrequency ablation
RFS: Recurrence-free survival
TACE: Transcatheter arterial chemoembolization
